# After-War Developments and Changes

**Published:** 1919-12-06

**Authors:** 


### THE LONDON HOSPITAL.

### After-War Developments and Changes.

The Committee of the London Hospital had hoped that on the conclusion of war they would have been able to proceed with building a doctors' hostel on a site closely adjoining the hospital. Before the war the estimated cost was £60,000; it is now £149,000, and, with very great regret, this work must be postponed on account of lack of funds. The necessity is a great misfortune to the hospital, and certain very important improvements and enlargements of certain laboratories are all held up for the same reason.

### The New Annexe at Reigate.

The Home at Reigate, which is to be known and used as an annexe of the hospital, a gift of the executors of the late W. C. Alexander, has now been opened, and the first patients have been sent. More of such annexes are needed in order that patients who now, on an average, occupy beds in London for twenty-one days, could be sent to the fresh air in the country after the first week. Their recovery would be more speedy and the hospital would be able to admit twice the number of patients. Moreover, heavy standing charges, such as cost of the bacteriological, clinical, *x*-ray and other laboratories, would be available for double the number of patients. Such a scheme is recognised as more urgent when one is informed that there are no fewer than 729 patients waiting for admission.

Mrs. Croft most generously has promised £1,000, and has already sent £500 towards the cost of upkeep of these twenty beds at Reigate.

### Preparations for Influenza.

The Committee and medical staff of the hospital have made all preparatory arrangements for a possible recurrence of the influenza epidemic, although the best scientific opinion is understood to be against the probability of a return, on the grounds that when serious epidemics have occurred before (and it is about thirty years since the last) it was found that the germ to which the disease was due seemed to lose its virulence in the course of about eighteen months. It is possible that there may be a slight recurrence in February, but by no means of such severity as the last. Cases of influenza treated recently at the London Hospital have quickly recovered.

A special section of the out-patient department will be opened in January under the care of Dr. Miller for the treatment of ailments in children. Such a department has always been needed, and it was never more wanted than now. The Committee expect it to grow rapidly.

### New Secretarial Appointment.

Hitherto the House Governor has held the joint appointment of House Governor and Secretary, but the work has so grown that the Committee have felt that the appointment should be divided, and Mr. Arthur G. Elliott has been appointed Secretary of the hospital, Mr. Morris retaining the appointment as House Governor.

